# AI energized hydrogel design, optimization and application in biomedicine

**DOI:** 10.1016/j.mtbio.2024.101014

**Published:** 2024-02-29

**Authors:** Zuhao Li, Peiran Song, Guangfeng Li, Yafei Han, Xiaoxiang Ren, Long Bai, Jiacan Su

**Affiliations:** a Department of Orthopedics, Xinhua Hospital Affiliated to Shanghai Jiao Tong University School of Medicine, Shanghai, 200092, China; b Organoid Research Center, Institute of Translational Medicine, Shanghai University, Shanghai, 200444, China; c National Center for Translational Medicine (Shanghai) SHU Branch, Shanghai University, Shanghai, 200444, China

**Keywords:** Artificial intelligence, Hydrogel, Design, Optimization, Biomedicine application

## Abstract

Traditional hydrogel design and optimization methods usually rely on repeated experiments, which is time-consuming and expensive, resulting in a slow-moving of advanced hydrogel development. With the rapid development of artificial intelligence (AI) technology and increasing material data, AI-energized design and optimization of hydrogels for biomedical applications has emerged as a revolutionary breakthrough in materials science. This review begins by outlining the history of AI and the potential advantages of using AI in the design and optimization of hydrogels, such as prediction and optimization of properties, multi-attribute optimization, high-throughput screening, automated material discovery, optimizing experimental design, and *etc*. Then, we focus on the various applications of hydrogels supported by AI technology in biomedicine, including drug delivery, bio-inks for advanced manufacturing, tissue repair, and biosensors, so as to provide a clear and comprehensive understanding of researchers in this field. Finally, we discuss the future directions and prospects, and provide a new perspective for the research and development of novel hydrogel materials for biomedical applications.

## Graphical abstract

**Figure:**
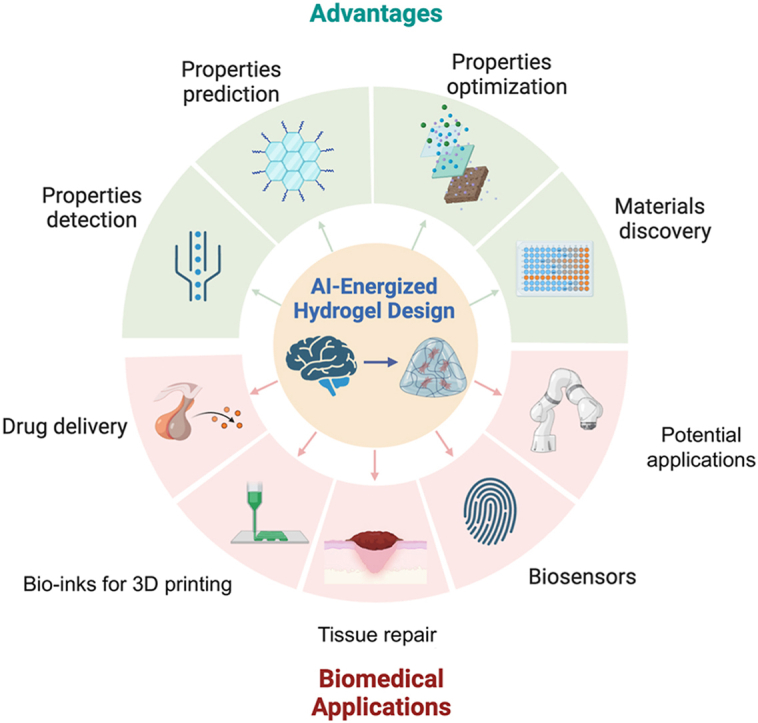

## 1 Introduction

With the tremendous progress made in materials synthesis technology, biomaterials have become the foundation for numerous emerging pharmaceutical and medical applications. They are also a critical issue of research in addressing biomedical needs [[1], [2], [3], [4]]. Particularly, hydrogels are a type of three-dimensional (3D) network structure primarily composed of water and network polymers. These materials exhibit high water content and have similarities to natural tissues [5,6]. Hydrogels have been greatly developed and optimized in recent decades due to a variety of fascinating physical and chemical properties, including biocompatibility, high water absorption, degradability, injectable properties, adjustable mechanical properties, and intelligent environmental responsiveness. As a result, they have found broad applications in drug delivery system, wound dressing, contact lens, bone tissue engineering, soft robotics, biosensors and *etc* [[7], [8], [9]]. However, the development of hydrogels is hampered by various challenges such as complicated artificial design process, time-consuming post-optimization, and long application cycles [10]. Addressing these problems requires a comprehensive understanding and application of interdisciplinary techniques, combined with advanced experimental techniques and computational simulation methods.

Artificial intelligence (AI) is a discipline that focuses on enabling computers to think, judge, and make decisions like humans. AI systems can undergo training using extensive datasets to make predictions, categorize objects, and carry out various intricate tasks. This field encompasses sub-fields such as machine learning (ML), natural language processing, images recognition, and algorithms and models that find wide applications across various industries [11,12]. ML, as an advanced and innovative field stemming from the advancements in AI, is a multidisciplinary domain that integrates control theory, computer science, determinism, mathematics, philosophy, and various other disciplines. Its intersection with these diverse fields makes it a dynamic and all-encompassing area of study [13,14]. ML involves the study, simulation, and implementation of human learning activities using computers. This interdisciplinary field focuses on developing algorithms and models that enable computers to learn from data and improve their performance over time [15]. The use of AI techniques, for example, high-throughput experimentation and the expansion of results to the database of Food and Drug Administration (FDA)-approved excipients, have greatly enhanced the progress of biomaterial design and production [16]. Incorporating ML techniques has further expedited biomaterial synthesis by shifting it towards a data-driven paradigm, leveraging descriptive-predictive-prescriptive methods and large-scale data analysis to optimize the search for the most effective biomaterials [17]. Specifically, the application of AI in the design and preparation of hydrogels offers numerous potential advantages. For instance, AI technology allows for the prediction and optimization of hydrogel composition and properties. Models can be built to automatically adjust parameters during hydrogel preparation process to achieve the best preparation outcomes [18,19]. Additionally, AI also has the potential to significantly impact the application of hydrogels. In the biomedical field, for example, hydrogels are often used for tissue engineering, drug delivery systems, and wound dressings. AI's image processing and recognition capabilities can automatically analyze and diagnose a patient's wound or lesion area, subsequently guiding the selection of suitable hydrogel materials and preparation methods. Furthermore, AI can be employed to monitor hydrogel performance and application environment. By utilizing sensor networks and data acquisition systems, real-time monitoring of parameters such as temperature, humidity, and pH value can be achieved. Combined with AI algorithms, any deviations from normal conditions can be promptly identified, enabling appropriate actions to ensure hydrogel performance and stability [[20], [21], [22], [23]].

Overall, the application of AI in hydrogels encompasses various aspects, including material design and preparation process optimization, material characterization analysis, high-throughput screening, and performance monitoring and control. These applications facilitate the improvement the function and application effectiveness of hydrogels, thereby promoting advancements in hydrogel technology. Although AI has begun to applied to the design and optimization of hydrogels and their biomedical applications, as far as we know, there is currently no comprehensive review that summarizes the integration of AI and hydrogels. In this review, the evolution of AI and its benefits in the context of hydrogels are briefly outlined, along with its application scenarios. Subsequently, we provide a comprehensive description of the state-of-the-art progress in the field of AI-energized hydrogel design and optimization, and its applications in biomedicine. Finally, we will present a discussion of the prospects and limitations of AI-Energized hydrogels. Through comprehensive research and analysis of relevant literature, our goal is to offer a clear and deep understanding for researchers and pave the way for future study in this field.

## 2 Overview of hydrogels and its development dilemma

**Fig. 1:**
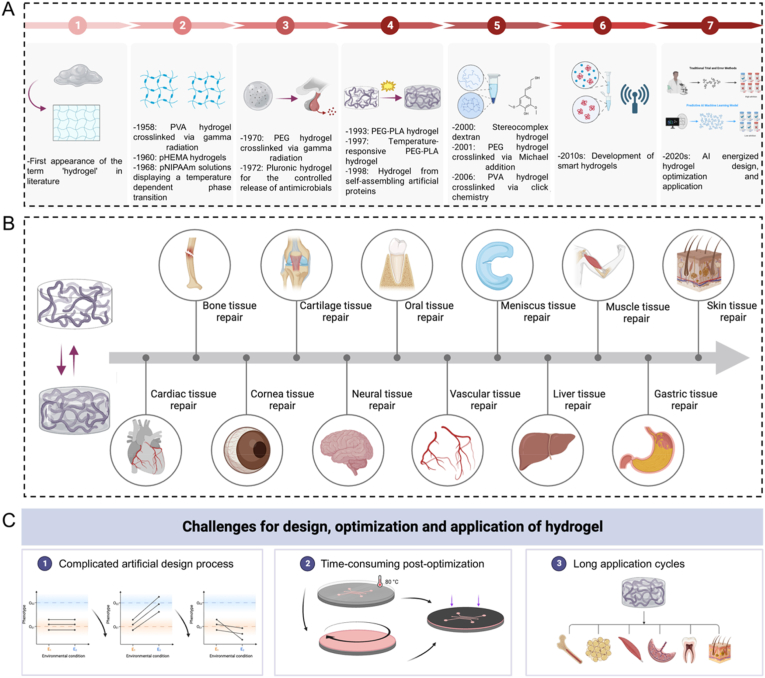
(A) The development history of hydrogels. (B) Hydrogels for various tissue engineering applications, such as repair the tissues of bone, cartilage, oral, meniscus, muscle, skin, cardiac, cornea, neural, vascular, hepatic, gastric, and so on. (C) Problems and challenges in the design, optimization and biomedical applications of hydrogels.

Since the pioneering work of Wichterle et al. in utilizing hydrated hydroxymethyl methacrylate (HEMA) network in contact lenses in the 1960s, various functional hydrogels have sparked significant research interest in the application of tissue engineering and biomedicine (Fig. 1A) [24,25]. Due to the presence of hydrophilic components within the polymer backbone, hydrogels have the ability to retain a significant amount of water and exhibit physicochemical properties resembling those of liquid water. This unique property allows hydrogels to display a solid-like rheological behavior on a macroscopic scale. This fascinating characteristic of hydrogels opens up the possibility of mimicking various features of the ECM found in tissues [26]. Furthermore, the gelation of hydrogels can be achieved through different methods such as physical cross-linking, dynamic cross-linking, and chemical cross-linking [27]. These techniques offer opportunities for engineering the gelation process to meet specific needs. Additionally, advanced chemical approaches have been developed to precisely manipulate the shape, structure, and architecture of hydrogels. Through these methods, hydrogels can be tailored with desirable functionalities including adjustable properties, excellent biocompatibility, controllable degradability, and mechanical compatibility with biological tissues. This unique characteristic makes them similar to biological tissues, enabling interactions with living cells and surrounding environments [28]. As a result, they have found broad applications in drug delivery system, wound dressing, contact lens, bone tissue engineering, biosensors and *etc* (Fig. 1B) [6,[29], [30], [31], [32], [33], [34]]. Details of current preparation, performance and application of typical hydrogels are listed in Table 1 [[35], [36], [37], [38], [39], [40], [41], [42]].

**Table 1:** Details of preparation, performance and application of typical hydrogels.

Hydrogels	Preparation	Performance	Application	Refs
Polyacrylamide (PAAm)	Free radical polymerization of acrylamide monomers in the presence of a crosslinker, such as MBA.	High water absorption capacity and tunable mechanical strength, depending on the crosslinking density.	Tissuee engineering, drug delivery, wound dressings, and etc. due to their biocompatibility and ability to swell in biological fluids.	[35]
Polyvinyl Alcohol (PVA)	Crosslinking PVA chains with a crosslinking agent, such as glutaraldehyde or borate ions.	Excellent biocompatibility, high water content, and good mechanical properties.	Contact lenses, wound dressing materials, and as scaffolds for tissue engineering due to their biocompatibility and optical clarity.	[36,37]
Polyethylene Glycol (PEG)	Physical or chemical crosslinking of PEG chains with crosslinking agents or through photopolymerization.	Excellent tunability of mechanical properties and degradation rates.	3D cell culture platforms, injectable materials for tissue engineering, and drug delivery systems.	[38,39]
Sodium Alginate	Ionotropic gelation of sodium alginate with divalent cations, such as calcium ions.	High water absorption capacity and can form a gel under mild conditions	Pharmaceutical industry for drug delivery, food industry for encapsulation of bioactive compounds.	[[40], [41], [42]]

In tissue engineering, hydrogels provide a supportive framework for the growth and regeneration of cells. Their biocompatibility and ability to mimic the natural ECM make them ideal scaffolds for tissue regeneration. By encapsulating cells within hydrogel matrices, scientists have successfully created artificial tissues and organs, leading to advancements in regenerative medicine [43,44]. Beyond tissue engineering, hydrogels have also found applications in drug delivery systems. The porous structure of hydrogels allows for controlled release of drugs, ensuring their gradual release over an extended period. This controlled drug delivery minimizes side effects and enhances therapeutic efficacy. Furthermore, hydrogels can be tailored to respond to specific stimuli, such as temperature, pH, or enzymatic activity, enabling targeted drug delivery and precise treatment [[45], [46], [47]]. Moreover, hydrogels have been explored for biosensing and diagnostic purposes. By incorporating specific molecules or nanoparticles into the hydrogel network, researchers have developed sensors capable of detecting various analytes, including glucose, proteins, and DNA. These hydrogel-based biosensors offer a sensitive and selective detection platform for disease diagnosis and monitoring [48,49].

However, the development of hydrogels, as well as biomaterials in general, has been hampered by challenges such as complicated artificial design process, time-consuming post-optimization, and prolonged preclinical testing cycles. These challenges include material screening, control of the preparation process, time and cost constraints, complex testing, characterization, optimization and prediction [10,50]. The design and preparation of hydrogels with specific properties often involve time-consuming trial and error processes to optimize the formulation (Fig. 1C). Overcoming these bottlenecks, finding effective optimization methods and predictive models to enhance hydrogel performance and preparation efficiency remain major challenges [51]. Addressing these problems requires the comprehensive application of materials science, chemical engineering, and other relevant fields of knowledge, combined with advanced experimental techniques and computational simulation methods. Predictably, with the continuous progress made in interdisciplinary, these bottlenecks are expected to be gradually overcome.

## 3 Overview of AI and its prospects in hydrogels

AI, originating in computer science, involves the development of machines with the ability to simulate human intelligence. These intelligent systems are programmed to think and learn in a manner similar to humans. They are designed to carry out tasks that traditionally necessitate human intelligence, including language translation, speech recognition, problem-solving, and decision-making [52]. The AI systems use algorithms and data to analyze and interpret information, make predictions, and take actions based on their analysis. AI technology is widely applicable and is used across a broad spectrum of industries, including medical care, finance, industrial manufacturing, transportation, and many other domains. Its versatile applications have made it an essential tool in modern society [53]. The origins of AI can be dated back to the 1950s [54], a time when John McCarthy first introduced the term "AI" in 1956 during a conference at Dartmouth College [55]. Over the past few decades, AI has gone through multiple stages and significant milestones. Here are some important developments in its history:

**Symbolic Period (1956**–**1974)**: During this period, the focus was mainly on using logical symbols and rules to represent knowledge and reasoning. Many symbol-based expert systems emerged during this time.

**Connectionist Period (1980s)**: Connectionism emphasized simulating the connections and learning processes between neurons in the human brain through network structures. This period witnessed the rise of research on neural networks and deep learning.

**Knowledge-based Period (1980**–**1995)**: The knowledge-based period focused on constructing systems with a large amount of domain-specific knowledge. Expert systems and rule-based reasoning became mainstream.

**Statistical Learning Period (1995-present)**: With the improvement in computational power and the widespread application of big data, statistical learning methods, for instance, support vector machines, random forests, and deep learning have become the primary approaches. This period is also known as the “era of machine learning”.

**Reinforcement Learning Period (2006-present)**: Reinforcement learning is a method that allows intelligent agents to learn through trial and error and feedback. This approach has found extensive applications in fields such as gaming and robot control.

Recent milestones, such as AlphaGo [56], AlphaFold [57], and ChatGPT [58], have permeated various industries and achieved significant breakthroughs, and its potential for applications has an extremely vast space limited only by our imagination. In the future, AI will continue to develop rapidly, bringing more convenience and innovation to our lives and work.

**Fig. 2:**
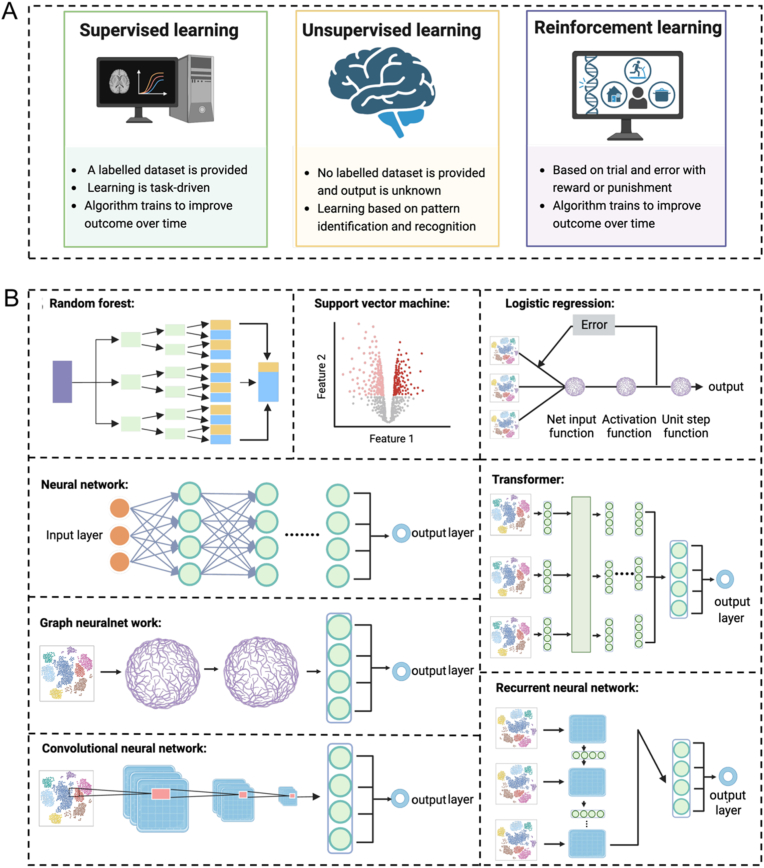
(A) The main types of ML, including supervised learning, unsupervised learning, and reinforcement learning. (B) Several common ML models offer the tools and methodologies for AI systems.

ML, as a subset of AI, which has emerged as the most advanced technology stemming from the progress of AI, forms the fundamental basis for the majority of AI applications in the present era. ML encompasses three primary strategies: supervised learning, unsupervised learning, and reinforcement learning (Fig. 2A) [59,60]. There are several common ML models, such as random forests (RF), support vector machines (SVM), logistic regression, neural networks, recurrent neural networks (RNNs), convolutional neural networks (CNNs), graph neural networks (GNNs), and transformers, offer the tools and methodologies that enable AI systems to accomplish complex tasks that were once considered unattainable (Fig. 2B). As described in our previous review, each different strategy and ML model has its own strengths and fields of application that can be used to solve different problems [59].

AI technology offers numerous advantages in the design and optimization of hydrogels. For materials property prediction and design, AI is used to process extensive volumes of data and train models to predict the physical and chemical properties of different hydrogel materials. This helps researchers in designing customized hydrogel materials to meet specific application requirements. For optimization, through ML algorithms, AI can analyze the relationships between the composition, structure, and properties of hydrogels, providing optimization recommendations. It assists researchers in quickly screening the best material combinations to enhance the performance and stability of hydrogels. AI-energized hydrogel products have various potential application scenarios in biomedical field, including wound healing, tissue engineering, advanced manufacturing, drug delivery, and biosensors. AI can assist in designing and manufacturing these hydrogel materials, improving the efficiency and safety of the products. In summary, the application of AI in hydrogel design and optimization accelerates the material development process, enhances material properties, and promotes the use of hydrogels in various fields.

## 4 Advantages of AI in hydrogel engineering

Owing to various fascinating physical and chemical properties, hydrogels have widely applied in drug delivery system, wound dressing, contact lens, tissue engineering, and *etc* [32,[61], [62], [63], [64], [65], [66]]. However, the developmental workflow of hydrogels, as well as biomaterials in general, has remained slow-moving due to some bottlenecks in the design and optimization of hydrogels. With the rapid development of AI technology, there are many potential advantages in the design and optimization of materials, such as prediction and optimization of properties, multi-attribute optimization, high-throughput screening, automated material discovery, optimizing experimental design, and *etc*. Herein, we describe some potentially important advantages of AI in hydrogel design and optimization in hopes of providing ideas for the preparation of advanced hydrogels.

### 4.1 Detecting the properties of hydrogels

Investigating each hydrogel formulation in practice is both a costly and time-consuming endeavor. In some cases, some formulations aren't feasible due to their material properties, and inspecting them wastes valuable resources. Computational prediction naturally plays a critical role in the optimization of hydrogel formulations [60]. For the past few years, AI has obtained significant attention in the area of material characterization analysis. Deep learning, in particular, is highly effective in addressing complex nonlinear mapping [67,68].

**Fig. 3:**
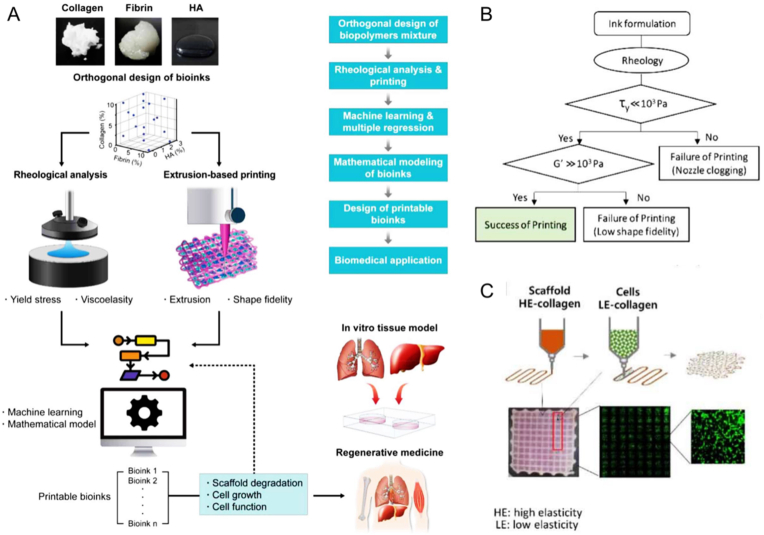
(A) Illustration of bio-inks development according to mathematical strategies to predict tissue engineering issues. (B) Schematic diagram for developing 3D printable naturally derived bio-inks. (C) Scheme illustrating the preparation of the cell-laden 3D biomimetic structure composed by low viscosity hydrogel (1% collagen) as cell vehicles and high viscosity hydrogel bio-inks as frameworks. Adapted with permission [73]. Copyright © 2020, IOP Publishing Ltd.

The potential applications of flexible hydrogels as pressure distribution sensors are indeed promising. Whereas the existing hydrogel pressure distribution sensors employ an array-type structure with intricate wiring and exhibit exceedingly low resolution, significantly impeding the flexibility of the hydrogels and constraining its potential development and applications [69,70]. To address these limitations, Liu et al. developed a pressure distribution reconstruction model according to the hydrogel pressure distribution sensors using a ML method. To enhance the accuracy of plantar pressure distribution reconstruction, a substantial quantity of plantar pressure distribution data collected from clinical sources can be fed into the network for secondary learning [71]. The authors utilize the ML method due to its capacity to conduct targeted optimization and secondary learning, which are important advantages compared to the traditional electrical impedance tomography method. A comprehensive understanding of the rheological properties of polymeric materials can be harnessed to design a diverse range of injectable hydrogels, including those used as bio-inks for 3D printing. Furthermore, advanced techniques have utilized AI-energized methods to detect the viscosity of polymeric materials [72]. In a recent study, an inductive logic programming-based method was employed to assess the correlation between the rheological behaviors and printability of FDA-approved natural polymers (Fig. 3) [73]. This powerful programming tool, along with other ML strategies, is categorized under the subset of AI, where advanced computational models are capable of processing massive amounts of data to recognize patterns [74]. However, as the field of AI continues to evolve, it becomes increasingly important to conduct new studies focused on predicting the rheological behaviors of prepared hydrogels. For detecting the properties of prepared hydrogels, AI can construct a complex composition-process-structure-property model to analyze the mechanical properties, water absorption properties, viscosity, stability, conductivity, degradability, and other inherent characteristics of prepared hydrogels. Consequently, efficient characterization analysis energized by AI offers a convenient procedure for the design and optimization of hydrogels.

### 4.2 Predicting and optimizing the properties of hydrogels

**Fig. 4:**
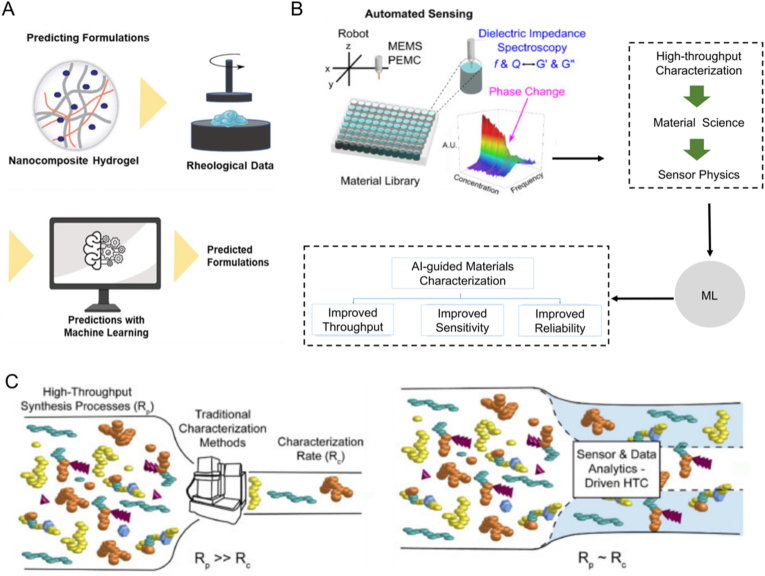
(A) Diagram of optimizing hydrogel formulations by using ML. Rheological information of hydrogels must be first collected through experiments or obtained from literature. Then, the collected information is fed into ML-based algorithms. Robust algorithms can predict the properties of different components and help optimize the formulation of hydrogels. **(**B) Illustration of a method for rapidly and autonomously characterizing the rheological properties of hydrogels by high-throughput through automated sensing and physically guided supervised ML. Adapted with permission [76]. Copyright © 2022, Elsevier Ltd. (C) The impact of innovative autonomous sensors and data-driven HTC strategies on achievable screening throughput compared to conventional characterization methods. Adapted with permission [76]. Copyright © 2022, Elsevier Ltd.

High-throughput characterization of the relations between composition, process, structure, and property is essential in facilitating the discovery of molecules and materials, as well as the formation of manufacturing paradigms. For predicting and optimizing the properties of hydrogels, AI can analyze the complex composition-process-structure-property model to predict various properties like mechanical strength, water absorption, stability, and etc., thus providing guidance for hydrogel design and optimization. For example, a comprehensive understanding of the rheological properties of polymeric materials can be leveraged to fabricate injectable hydrogels suitable for 3D printing. ML can accurately predict the rheological properties of hydrogels and optimize their formulations (Fig. 4A). Recently, a robust supervised ML model successfully predicted the viscosity of polymer composites containing nanoparticles [75]. By utilizing automatic sensing and physical guidance of ML, the rheological properties of hydrogels can be rapidly and autonomously characterized with high-throughput (Fig. 4B). Specifically, this innovative high-throughput method accurately characterized the rheological properties of hydrogels in 96-well plates, achieving a remarkable rate of 24 s/sample, which is 70 times faster than the current state-of-the-art [76]. Ultimately, this high-throughput performance prediction strategy paves the way for optimal design of hydrogels.

In addition to monitoring the rheological properties of hydrogels, AI-energized high-throughput screening can also predict and optimize various properties of hydrogels. A statistical and AI method based on principal component analysis can be employed to evaluate the adsorption efficiency of hydrogels by applying varying parameters, achieving a rapid and efficient evaluation of the composite hydrogels' properties [77].

The influence of critical parameters such as raw materials concentrations size and zeta potential of nanoparticles, and pH value of solutions on the stiffness, gelation time, and adhesion behavior can be comprehensively investigated by an ANNs model, thus predicting the properties of hydrogels [[78], [79], [80]]. Furthermore, AI-energized high-throughput screening significantly reduces raw material consumption. Seifermann et al. reported a high-throughput tactics according to miniaturized experiments and ML to optimize the photostability properties of materials. Within 13 experiments, the authors successfully optimized the material properties of approximately 13,440 possible hydrogels, using a total of only 0.65 mL of the original solution and about 170 mg, that is, 836 μmol monomer and crosslinker [81].

Overall, utilizing AI technology to predict and optimize the properties of prepared hydrogels significantly decreases the time and expenses associated with individual experiments. The AI-energized high-throughput screening strategy has demonstrated notable efficiency and practicality compared to traditional methods for predicting and optimizing properties of hydrogels. It is anticipated to emerge as a novel tool for quality control and assurance in emerging sectors.

### 4.3 Materials discovery of hydrogels

**Fig. 5:**
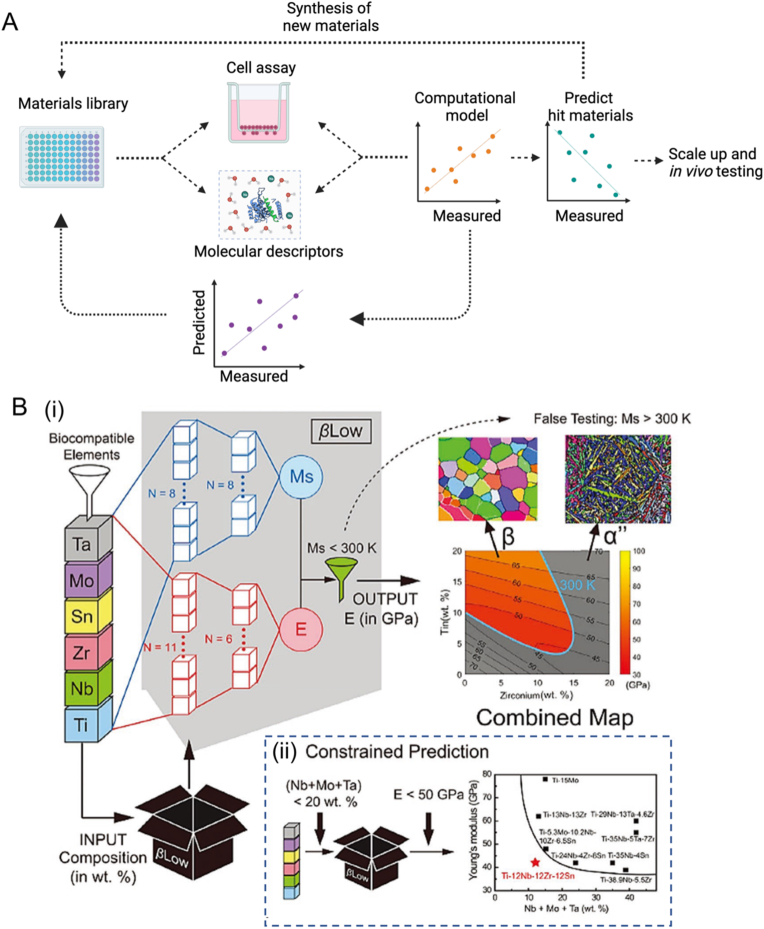
(A) Overview of the AI-energized materials discovery. Adapted from CC-BY open access publications [88]. Copyright © 2016, Elsevier Ltd. (B) An illustration of material discovery, using titanium alloy as an example. (i) Properties prediction processes, such as filtering of martensite start (Ms) temperature and combining maps. (ii) Constraining the prediction in βLow-assisted alloy development for low-modulus and low-β stabilizer β-titanium alloys. Adapted with permission [89]. Copyright © 2020, Elsevier Ltd.

AI-energized materials discovery is a cutting-edge field that combines AI algorithms and computational methods to expedite the process of identifying and designing novel materials with specific properties [82]. Thanks to advancements in materials science, there is a substantial amount of data available from experimental and simulation studies, which forms the basis for employing ML techniques in materials research and development [83,84]. AI algorithms can be applied in various ways within materials discovery. By analyzing and predicting the behavior of materials using the available data, AI can assist researchers in conducting more efficient and targeted experimentation [[85], [86], [87]]. Say concretely, this entails analyzing existing databases, scientific literature, and experimental results to uncover patterns and relationships between the composition, structure, and properties of materials (Fig. 5) [17,88,89]. Quantitative structure properties relationships provide the unique property to correlate microscale molecular descriptors to larger macroscale material properties [90,91]. Such knowledge can then guide the search for novel materials with desired characteristics.

The development of materials data science has spurred initiatives such as the Materials Genome Initiative (MGI) in the United States, which aims to accelerate research cycles and reduce costs through high-throughput computing, data-driven methods, and big data technologies [92]. ML, which falls within the realm of AI and data science, plays a crucial role in the MGI. ML entails constructing computers that enhance their performance autonomously by leveraging past experiences. It is a rapidly growing interdisciplinary field dominated by computer science and statistics, and is essential to AI and data science [[93], [94], [95]]. The advancement in ML has been propelled by the emergence of innovative learning algorithms and theoretical frameworks, as well as the increasing availability of online data and low-cost computation. ML methods are extensively used across various domains, including science, technology, and commerce, facilitating evidence-based decision-making in areas, for example, medical care, manufacturing, education, finance, law enforcement, and marketing [96].

Hybrid materials have been the subject of research for several decades; however, the complete understanding of their formation remains elusive, and the evolution of novel compounds largely depends on exploratory and trial-and-error synthesis [[97], [98], [99]]. Recently, simulation- and data-driven methods have emerged as an alternative to the traditional method of experimental trial-and-error. ML models, in particular, have shown promise in predicting the conditions necessary for the formation of new hybrid materials. In fact, these models have outperformed traditional human strategies and have been successful in predicting the formation conditions with a certain level of accuracy, achieving an 89 percent success rate [100]. The integration of high-throughput synthesis and evaluation means enables the elucidation of the structure-property relationship using a large volume of empirical data, facilitating the identification of potential target candidates for synthetic efforts [101,102].

In summary, AI-energized materials discovery leverages AI algorithms and computational methods to accelerate the identification and design of new materials. The wealth of data available and ML techniques help researchers analyze and predict material behavior, facilitating more efficient experimentation and guiding the search for desired material properties. However, there is still much work to be done regarding the AI strategy in predicting materials design for hydrogels in biomedicine. There are several methods available that can provide insights for guiding the discovery or design of next-generation materials. Simulation-based predictions can assist in identifying potential target candidates for synthetic endeavors by analyzing their physical properties. Integrated high-throughput synthesis and evaluation means allows for the determination of structure-property relationships from large experimental datasets. Additionally, clustering based on similar outcomes can provide valuable insights. These AI-driven approaches can greatly facilitate the discovery and design of next-generation materials in the field of hydrogel biomedicine [103].

## 5 Applications of AI-energized hydrogel design in biomedicine

Ai-energized design and optimization can obtain advanced hydrogels, making them play a broader role in biomedical applications, for instance, drug delivery systems, bio-inks for advanced manufacturing, tissue repair, biosensors, and etc. Herein, we focus on the various applications of hydrogels supported by AI technology in biomedicine, so as to provide a clear and comprehensive understanding of researchers in this field.

### 5.1 Drug delivery systems

**Fig. 6:**
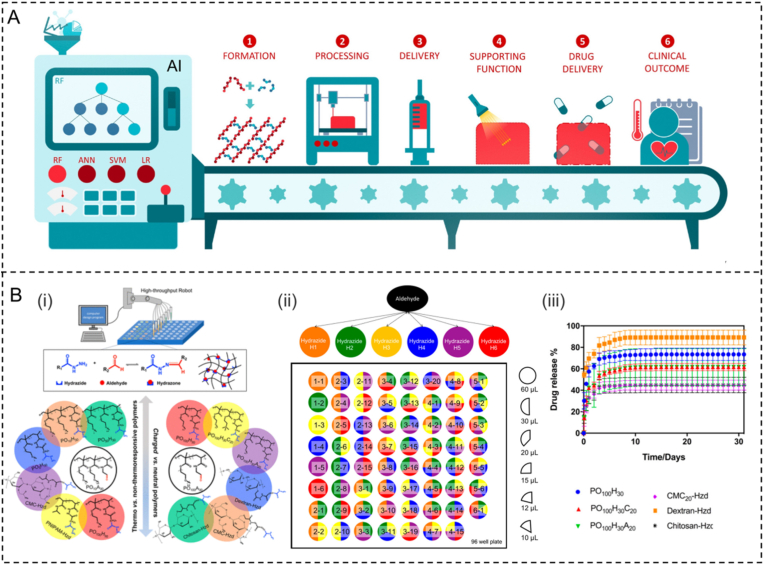
(A) AI strategies, such as RF, ANNs, and SVM, have been applied at multiple steps, including (1) forecasting hydrogel formation according to previous ingredients, (2) improving 3D printing performance, (3) adjusting injectable properties, (4) optimizing supporting functions, (5) optimizing and forecasting drug release curves, and (6) upgrading clinical effects, to enhance the preparation of hydrogel drug delivery systems. Adapted with permission [108]. Copyright © 2022, Elsevier Ltd. (B) Synthesis, analysis and optimization of specific injectable hydrogels for delivering proteins by the high-throughput strategies. Adapted with permission [119]. Copyright © 2019 American Chemical Society.

Hydrogels hold great promise as drug delivery systems, thanks to their excellent biocompatibility and similar properties to natural tissues [[104], [105], [106]]. However, the development workflow for hydrogel-based drug delivery systems relies heavily on trial and error, leading to significant requirements in terms of time and material resources. AI has the potential to mitigate this challenge by leveraging insights gained from collected data, enabling a more focused and predictive experimental method [107]. The applicability of AI in the workflow of hydrogels as drug delivery systems has been demonstrated through the establishment of predictive models, optimization of algorithms, and image processing and recognition. AI can be effectively utilized to predict hydrogel formation, optimize hydrogel performances, and ultimately to tune the drug release profiles (Fig. 6A) [108].

Firstly, AI technology is utilized to construct models for predicting the release behavior of drugs incorporated in hydrogels, considering the permeability, diffusion rate, dissociation rate, as well as the structure and properties of hydrogels. By analyzing a significant amount of experimental data, these models can provide insights into the release rate and duration of drugs in hydrogels, thus supporting the design of sustained-release drug systems [109,110]. Secondly, AI can enhance the performance and efficacy of drug sustained-release systems by optimizing algorithms. Through AI algorithms, the optimal drug loading, hydrogel composition ratio, preparation conditions, and other parameters can be determined to achieve prolonged or precisely controlled release rates. The optimization algorithm based on AI strategy can undergo multiple simulations and employ genetic algorithms to identify the optimal strategy for sustaining drug release [111,112]. Additionally, AI can facilitate the monitoring and control of drug slow-release systems through image processing and recognition technology [113,114]. For instance, by incorporating nanoparticles or markers to the surface or inside of the hydrogels, AI's image recognition capabilities enable real-time monitoring of the drug release profiles. This real-time data analysis allows for a deeper understanding of drug release, leading to more accurate adjustments of system parameters for better control of the drug release profiles [108,115].

Accurately predicting the formation of hydrogels is essential in the development of efficient drug delivery systems. To address this issue, Li et al. employed a combinatorial chemistry approach to construct a diverse library of >2000 peptides for the analysis of their self-assembly behavior [116]. In this study, the authors utilized AI to establish correlations between quantitative structure-property relationships, chemical properties, and self-assembly behaviors. Through this approach, the study identified prominent structural features successfully that significantly contribute to the formation of hydrogels.

After being successfully formed, the hydrogel system must have properties that are specifically designed for its intended purpose. These properties include the ability to respond to temperature changes for injectable delivery and interact with biological systems, such as a new mucoadhesive thermos-gelling hydrogel for sublingual enhancement [112] and orotransmucosal vaccine-delivery platforms [117]. For instance, Rio et al. utilized AI as an instrument to investigate interactions between polymers in order to develop thermosensitive hydrogels that are appropriate for delivering proteins to the rectum in cases of inflammatory bowel disease. Enemas containing Pluronic F68, Pluronic F127, and Methocel K4M were developed and analyzed for delivering proteins to the rectum. The researchers utilized a commercially available hybrid AI tool platform that integrated artificial neural networks (ANNs), fuzzy logic technologies (FormRules version 4.03), with the polymer concentrations as input variables to establish correlations with the ultimate characteristics of the hydrogels [118]. Through this AI-energized strategy, it is possible to determine the role of each polymer component in the hydrogel formulation on the features of the obtained hydrogel, for instance, F127 affects the injectability and mucosal adhesion.

**Fig. 7:**
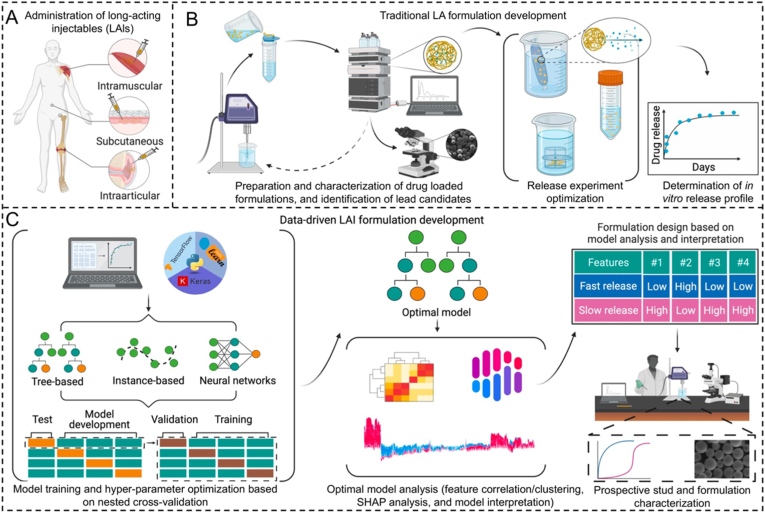
(A) Selected administration routes of long-acting injectables formulations approved by FDA. (B) Typical trial-and-error loop conventionally used in the development of classical long-acting injectables formulation. (C) Training and analyzing ML models to enhance the developing of new long-acting injectables systems, termed “Data-driven long-acting injectables formulation development”. Adapted with permission [122]. This is an open access article distributed under the terms of the Creative Commons CC BY license.

Achieving sustained and dose-specific release profiles is clinically significant for drug delivery systems and requires predicting and optimizing parameters, including minimizing uncontrol release and maximizing cumulative release [120,121]. But the compatibility of data-hungry ML techniques is limited by the time- and resource-intensive nature of drug release tests. To address this issue, a research pointed fabricating various hydrazine-crosslinked in situ formed hydrogels (126 in quadruplicate) using combinatorial methods. The initial characterization was completed within hours, followed by parallelized release experiments conducted in a 96-well plate format (Fig. 6B). The release curves were subsequently fitted to an amendatory first-order equation in order to determine the parameters associated with burst release, cumulative protein release, as well as release rate. The authors developed a partial least-squares model to explain 60–80% of the variance and forecast optimal polymer combinations for protein delivery applications [119]. In addition, Castro et al. developed a literature-mined dataset to estimate the critical parameters in regard to the formulation pipeline and dissolution characteristics in vitro [107]. Apart from these, it is also worth considering other relevant strategies used to the release systems. For instance, in a drug release research of injectable formulations, shapely additive explanation dependence plots were employed to evaluate characteristic importance and the interactions among the features. The resulting model achieved precise predictions of release using a small amount of training data consisting of 102 formulations. This achievement was further validated through an external dataset of 79 unseen release profiles, obtained from literature mining (Fig. 7) [122]. The authors pointed out that ML algorithms have the capability to predict experimental drug release from the advanced drug delivery systems. The use of these trained models can provide valuable insights for designing new long-acting injectables.

Long-acting injectable formulations are regarded as a strategy with great potential for treating chronic diseases due to their ability to enhance therapeutic efficacy, patient compliance, and safety. Moreover, previous studies have demonstrated the potential of utilizing AI algorithms to forecast release profiles from advanced drug delivery systems. These predictive models can effectively inform the development of novel long-acting injectable formulations. Employing this data-driven strategy promises to save time and economic budgets related to drug formulation development.

### 5.2 Bio-inks for advanced manufacturing

**Fig. 8:**
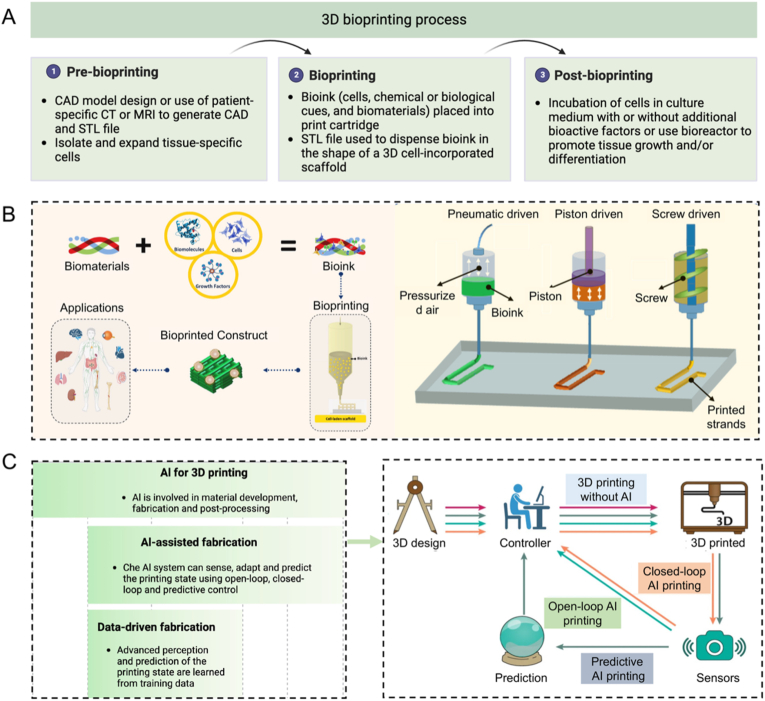
(A) The 3D bioprinting process includes pre-bioprinting, bioprinting, and post-bioprinting. (B) Constructs printed from bio-inks and applications and schematic of pneumatic-, piston-, and screw-driven printing. Adapted with permission [124]. Copyright © 2023 The Authors. Publishing services by Elsevier B.V. on behalf of KeAi Communications Co. Ltd. (C) AI can be involved in various stages of 3D bio-printing in different ways, including AI-energized fabrication and data-driven fabrication. The illustration indicates the bio-printing procedure for different levels AI involvement in fabrication, such as 3D printing without AI, open-loop AI printing, closed-loop AI printing and predictive AI printing. Adapted with permission [132]. Copyright © 2020, Springer Nature Limited.

Since the early 1980s, 3D printing technology has been in a booming phase, encompassing various techniques such as from fused-filament fabrication for complex plastic structures to the development of next-generation bioprinting technology (Fig. 8A) [123]. Hydrogels, as bio-inks to incorporate living cells and/or bioactive substances to form biomaterial solutions for printing 3D structured functional scaffolds in a layer-by-layer way. This technology is expected to play an important role in areas such as regenerative medicine, tissue repair and organ transplantation. The mechanical forces utilized in extrusion bioprinting can be categorized into three classes: pneumatic, piston, and screw-driven (Fig. 8B) [124]. Leveraging its intrinsic customizability and rapid fabricating capabilities, 3D bio-printing has the potential to facilitate the large-scale production of personalized artificial or bionic organs, as well as smart wearables [125,126]. Despite this potential, the development of 3D bio-printed multifunctional products is still at a preliminary stage. One significant challenge is formulating inks, typically hydrogels, that maintain functionality after printing and are compatible with other inks to fabricate complex multifunctional architectures [127]. Another obstacle arises from the fact that traditional printing platforms are chiefly ex situ printing [128]. The fabricate-then-transfer process has several drawbacks, including discrepancies between the printed and target surfaces, damage to delicate materials like hydrogels during manual handling, which can impact post-transfer fidelity, the risk of contamination during transportation and manual transplantation, and constraints in minimally invasive surgery [[129], [130], [131]]. A potential workaround is to employ AI-powered, minimally invasive 3D-printing methods to directly manufacture on target surfaces.

AI-powered printing utilizes past experiences to make predictions about future states, enabling quick adaptation to dynamic and changing targets [133]. In the realm of printing procedures, AI plays a prominent role at three levels: open-loop AI, closed-loop AI, and predictive AI (Fig. 8C). Detailed descriptions of the specific characteristics and advantages of these three AI-energized 3D printing strategies can be found in a previous review [132]. Herein, our focus is primarily on exploring the printability and advancements of these AI-energized hydrogels development and fabrication as bio-inks.

**Fig. 9:**
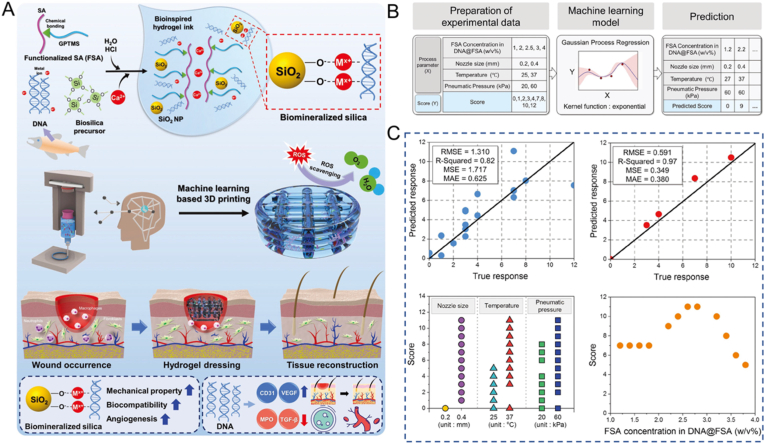
(A) Illustration of developing bioinspired 3D-printed hydrogels by DNA-induced biomineralization. (B) ML modeling applying Gaussian process regression to predict the printability score of the prepared hydrogel bio-inks. (C) Various scores based on variable nozzle size, temperature, pneumatic pressure, and FSA concentration. Adapted with permission [134]. This is an open access article distributed under the terms of the Creative Commons CC BY license.

In the realm of bio-inks design for 3D printing, the primary role of AI is to predict and optimize hydrogel properties, enable high-throughput screening, and facilitate new material discovery. Kim et al. utilized a ML based model system developed by MATLAB software to design a 3D-printable hydrogel ink composed of functionalized alginate and DNA (DNA@FSA inks) (Fig. 9). The printability scores of the datasets were predicted by adjusting the independent input variables, including printing temperature (27 °C and 37 °C), nozzle size (0.2 and 0.4 mm), pneumatic pressure (20 and 60 kPa), FSA concentration (from 1.2 to 3.8 w/v% at intervals of 0.2 w/v%), and gel concentration (1, 2, 2.5, 3, and 4 w/v%). The results revealed that the highest printability grade was obtained with FSA concentrations of 2.6 and 2.8 w/v%. Consequently, according to the ML approach, 3 w/v% FSA was used to optimize and stabilize the printing process [134]. The 3D-printed wound dressing was designed to achieve optimal porosity, allowing for effective absorption of exudate and blood at the wound site. Additionally, the mechanical properties of the dressing can be adjusted to ensure good shape fidelity and ease of printing during the 3D printing process. As a result, this innovative DNA-induced biomineralization strategy resulted in a functional platform with great potential for clinical applications in both acute and chronic wound repair.

Enhancing the quality of 3D printed bio-scaffolds extends beyond the accuracy and spatial control of material deposition. Preserving the functionality of the ink, particularly cell viability, throughout the printing process is crucial for achieving optimal print quality [135]. Given the distinct advantages of AI in material discovery and screening, and high-throughput prediction and optimization of material properties, it is anticipated that AI-energized bio-inks preparation will overcome the current technological barriers and pave the way for advancements in 3D printing technology.

### 5.3 Tissue repair

**Fig. 10:**
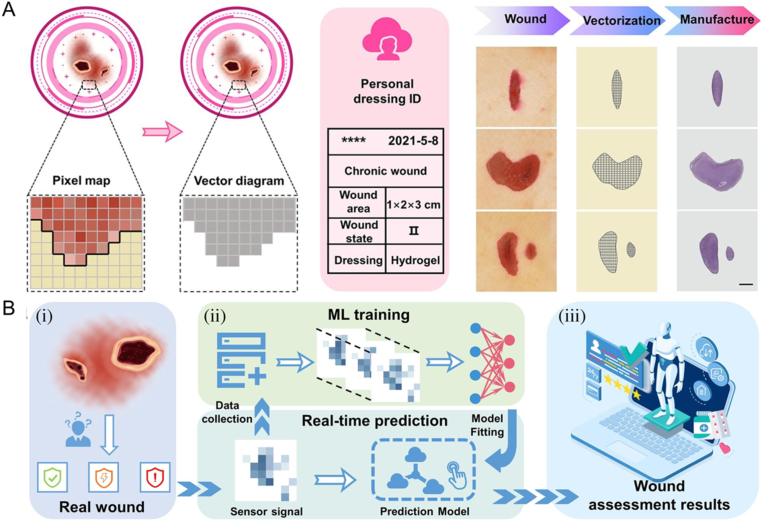
(A) Description of the wound recognition. Personalized 3D printed hydrogel wound dressings can match the shape and size of the wound by recognizing wound characteristics. (B) Flow chart of intelligent wound monitoring with multifunctional hydrogel dressings, including (i) wound recognition, (ii) real-time status supervising and (iii) customized wound management. Adapted with permission [143]. Copyright © 2022, Elsevier Ltd.

In recent years, a variety of tissue engineering strategies have been used to repair damaged tissues in the body, and great progress has been made [[136], [137], [138], [139]]. Hydrogels are commonly applied materials in repairing tissues, such as bone and skin tissues, and AI strategies can achieve more efficient and precise preparation [140]. AI can be utilized in various aspects of hydrogel composition screening, material preparation, and performance optimization. Through the analysis of extensive medical data and experiential knowledge, AI can assist in rapidly selecting appropriate material combinations and predict their performance through simulation and calculation. Additionally, AI can also employ ML algorithms to optimize the preparation process of hydrogels, thereby improving production efficiency and quality control [141,142]. For example, by using the image processing and recognition capabilities of AI, it is possible to automatically analyze and diagnose the wounds or lesion areas of patients, and then select appropriate hydrogel materials and preparation methods (Fig. 10A). AI-based algorithms can be employed to evaluate changes in wound characteristics, guiding the design and preparation of hydrogels, as well as estimating the efficacy of therapy and predicting healing outcomes (Fig. 10B) [143]. The multifunctional hydrogel wound dressing offers a comprehensive approach to wound care, combining precise treatment, real-time monitoring, and personalized management for intelligent wound monitoring. This innovative solution not only accelerates the healing process but also effectively reduces the risk of bacterial infections. It represents a significant advancement in the field of intelligent wound management and sets a new standard for future developments in this area.

**Fig. 11:**
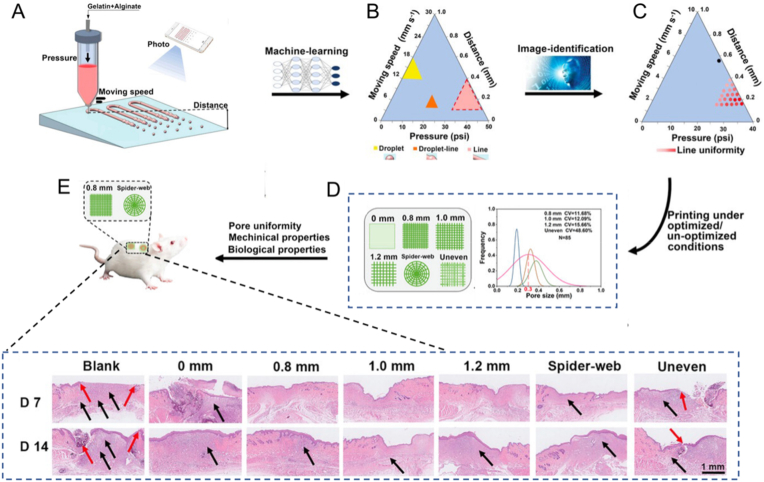
Illustration of the AI-HTPCSS for fast screening of the optimized extrusion bio-printing conditions of a given bio-printer and bio-ink combination. (A) Overview of the AI-HTPCSS. (B) The morphologies of extrusion patterns under different bio-printing parameters, such as droplets, lines of droplets, or lines. (C) Graphical representing the line uniformities of extruded patterns under different bio-printing parameters. (D) Optimized bio-printing conditions are sued to prepare multi-layer 3D mesh-like hydrogel scaffolds with different structures. (E) The application of the optimal printed hydrogel dressings for accelerating the healing of diabetic wounds. Adapted with permission [148]. Copyright © 2022, Wiley-VCH GmbH.

The application forms of hydrogels as wound dressings are various, among which hydrogels as bio-inks are used to prepare personalized dressings by 3D printing, which represents an advanced preparation method [[144], [145], [146]]. However, optimizing the printing parameters typically depends on previous knowledge and a significant number of laborious verification experiments [147]. To resolve the problem, a high-throughput printing-condition-screening system with the assistance of AI (AI-HTPCSS) was proposed by Chen et al., using extrusion bio-printing as the demonstration (Fig. 11A). Specifically, for the proof of concept, the phase diagram of alginate-gelatin ink for gel printing could be obtained based on AI-HTPCSS. (Fig. 11B and C). Based on the optimized conditions, 3D grid-like hydrogel scaffolds with different structures can be printed. The scaffolds exhibited good consistency with the digital models and possessed excellent mechanical and biological features (Fig. 11D). Finally, systematic in vitro and in vivo evaluations demonstrated that scaffolds printed under optimized conditions can apparently accelerate the diabetic wounds healing (Fig. 11E) [148]. The AI-HTPCSS proposed in this work demonstrated a universal platform for rapid screening of optimal printing conditions for given combinations of bio-printers and bio-ink materials, showing promise for potential applications in tissue engineering and regenerative medicine.

However, it should be noted that at the current level of technological development, hydrogels prepared using AI are still in the research and development stage. Before practical application, extensive clinical trials and approval processes are required to ensure their safety and effectiveness.

### 5.4 Biosensors

Hydrogels, as a kind of materials for preparing electronic skin and wearable devices as biosensors, have numerous advantages. For instance, hydrogels exhibit good flexibility and elasticity, allowing it to have better contact and adherence to human skin, thus increasing comfort when worn [149,150]. Hydrogels also possess excellent water absorption properties, which can help maintain the moisturization and stability of the skin. Additionally, hydrogels exhibit good conductivity, which can provide stable signal transmission and response [[151], [152], [153]]. As a potential candidate material for biosensors, the requirements for various properties of hydrogels are extremely demanding. However, relying on traditional material screening methods and optimization strategies greatly limits the design and preparation of advanced hydrogels, as the addition of more and more components during the material preparation process is required to enhance and regulate their mechanical, biomedical, electrical, and self-healing properties [[154], [155], [156]]. Because of its unique advantages in component screening, characterization analysis and performance optimization of hydrogels, AI is extremely important in the preparation of advanced electronic skin and wearable devices.

**Fig. 12:**
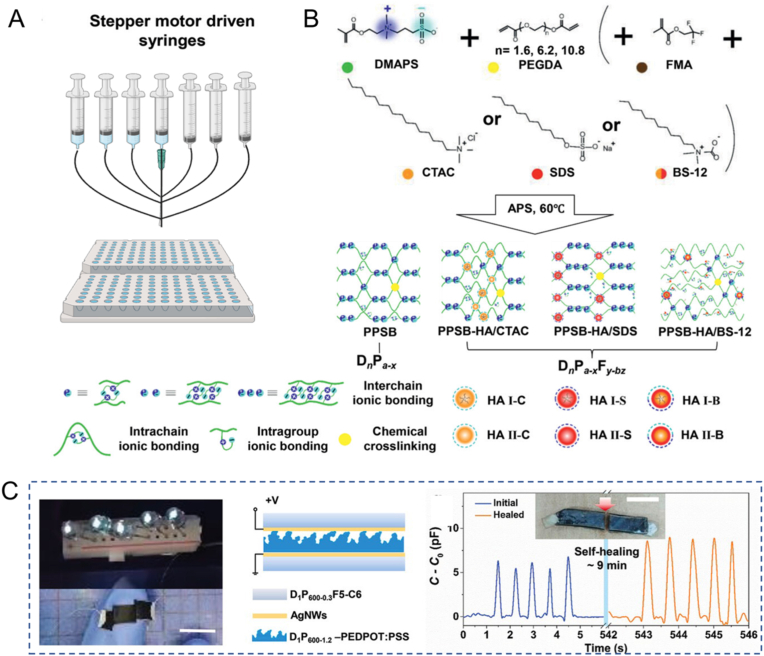
(A) Overview of high-throughput multi-channel feeder. (B) Schematic diagram of material synthesis reactions. (C) The device structure of the hydrogel-based capacitive sensor and its response to applied pressure before cutting and after self-healing are analyzed. Adapted with permission [157]. Copyright © 2021, Wiley-VCH GmbH.

For instance, high-throughput screening enables efficient and systematic exploration of the effects of multiple components on polysulfobetaine hydrogel properties, thus providing a valuable database for diverse applications. Through high-throughput screening, the authors obtained optimized polysulfobetaine hydrogels and demonstrated that this electronic skin processing exceptionally mechanical properties and self-healing capabilities at ambient conditions (Fig. 12) [157]. This work not only extends the high-throughput synthetic methodology to the field of hydrogel electronics but also opens up new avenues for healable flexible electronic devices through advancements in material development and device design.

By combining AI technology, electronic skin can achieve more intelligent functions. For example, by sensing environmental changes and physiological parameters of the human body, biosensors can monitor health conditions in real time and provide personalized health advice [158,159]. In addition, electronic skin can also communicate wirelessly with other devices or systems, enabling more convenient information exchange and control operations [160]. Through integration with ML module and proper training, the hydrogel-based platform achieved high accuracy in recognizing human handwriting movements, ranging from individual letters to words, phrases, and short sentences. This hydrogel-based ionic skin combines superior mechanical properties with self-evolving sensing capabilities, unleashing its potential as an intelligent human-device interface and promoting the application of AI in customized electronic devices [161].

It should be noted that further research and experimental validation are necessary for the development of this technology to ensure the stability, safety, and reliability of electronic skin. Moreover, considerations regarding personal privacy and data protection need to be taken into account during its application.

## 6 Summary and perspectives

Hydrogels are materials with a high-water content that exhibit unique properties, making them suitable for various applications [162]. However, the developmental workflow of hydrogels, which is largely based on trial and error and requires significant amounts of time and material resources, alike has remained slow-moving due to some bottlenecks in the design, optimization and application of hydrogels. AI has made significant advances in various fields, including the design and optimization of hydrogels for biomedical applications. AI can help alleviate this burden by obtaining insights from generated data to achieve more targeted and predictive experimental methods. AI-energized hydrogel design involves using ML algorithms to analyze large datasets and identify optimal combinations of polymers, cross-linking agents, and other additives to create hydrogels with specific properties. By leveraging AI, researchers can accelerate the process of designing and optimizing hydrogels with desired characteristics, such as mechanical strength, porosity, biocompatibility, and drug release profiles. Energized by AI, hydrogels have shown advanced applications in tissue engineering, including drug delivery, bio-inks for advanced manufacturing, tissue repair, and biosensors.

**Fig. 13:**
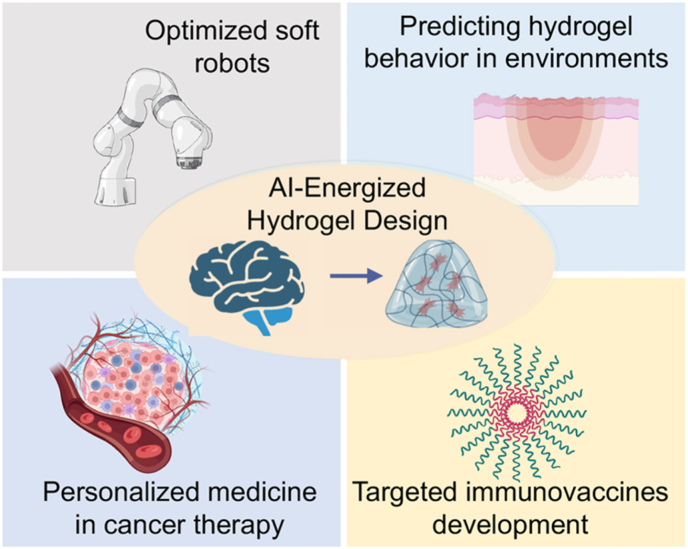
The current development trends and potential applications of AI-energized design and optimization of hydrogels in biomedicine.

Besides these already developed biomedical applications, AI-energized hydrogel design and optimization also has potential applications in a number of other fields (Fig. 13). For example, the utilization of ML-based methods shows promise for both soft robot proprioception and object recognition when resistive or capacitive readings are accessible [163,164]. The combination of recently developed physics engines and deep learning is utilized to optimize soft robots [165]. These gradient-based optimization methods have the potential to be more computationally efficient. In addition, through simulation of virtual robots using data-driven models, it becomes possible to concurrently optimize various objectives, including geometry, controller models, and physical system properties for system identification [166]. Using ML to assist in sensing and control holds great potential as it can expand the capabilities of intelligent robotics systems. In addition, AI-energized hydrogels also are expected to play an important role in personalized medicine with a focus on cancer therapy and immunovaccines. On the one hand, AI using ML algorithms to recognize and predict the pattern of various factors in the tumor microenvironment, including analyzing the gene expression pattern, protein level and cell type of tumor tissue, as well as predicting the tumor's response to different treatments. On the other hand, through the acquisition of these data, AI-energized design and optimization of hydrogels can better mimic and build 3D tumor microenvironments, and is expected to provide equal or even better practicality than animal models or other preclinical models.

One area where AI has been particularly useful is in predicting the behavior of hydrogels in complex biological environments [167]. Traditional methods for hydrogel design and optimization often rely on trial-and-error approaches, which can be time-consuming and costly. AI algorithms can analyze existing experimental data to identify patterns and correlations between hydrogel composition and performance. This information can then be used to predict the behavior of new hydrogel formulations and guide the design process. Furthermore, AI can also aid in the discovery of novel hydrogel materials by performing virtual screening of large databases, simulating molecular interactions, and predicting the properties of potential candidate molecules. This can help researchers identify promising candidates for further experimental testing, potentially saving time and resources. In addition to design and discovery, AI can also assist in optimizing the manufacturing process of hydrogels. By analyzing process parameters and experimental data, AI algorithms can help identify optimal conditions for hydrogel synthesis, resulting in improved reproducibility and scalability. In summary, AI offers valuable tools and techniques for the design and optimization of hydrogels. By leveraging ML, optimization algorithms, and generative models, AI can accelerate the development of advanced hydrogel materials with applications in various fields.

Despite this potential, the development of AI-energized multifunctional hydrogel products is still at a preliminary stage. The utilization of AI generally necessitates a larger volume of data for training, validation, and testing purposes. For example, in the development of hydrogel drug delivery system, in addition to effectively modulating the input parameters, it is crucial to have sufficiently informative output hydrogel release. The availability of abundant experimental data covering both the input parameters and output release would facilitate the development of enhanced predictive insights into the relationships. Therefore, there is a need to establish a centralized and standardized system for collating hydrogel drug release results, enabling effortless cross-comparisons between studies [[168], [169], [170]]. One potential avenue is the implementation of a standardized release and testing protocol that researchers can adopt in their investigations. Such a protocol would enable the normalization of initial parameters and variables across different research groups, facilitating more efficient comparisons. Moreover, standardization could be extended to data annotation and processing methods, ensuring the generation of a comprehensive hyperparametric dataset [171].

It is important to note that while AI-energized hydrogel design shows great promise, it is still an emerging field. Further research and development are needed to fully harness the potential of AI in designing hydrogels for biomedical applications. As technology continues to advance, we can expect AI to play an increasingly crucial role in accelerating the development of innovative hydrogel-based therapies.

## CRediT authorship contribution statement

**Zuhao Li:** Writing – original draft, Software, Investigation, Data curation, Conceptualization. **Peiran Song:** Resources, Methodology, Investigation, Formal analysis. **Guangfeng Li:** Visualization, Validation, Resources, Conceptualization. **Yafei Han:** Validation, Software, Methodology. **Xiaoxiang Ren:** Writing – review & editing, Supervision. **Long Bai:** Writing – review & editing, Supervision, Methodology. **Jiacan Su:** Writing – review & editing, Supervision, Project administration.

## Declaration of competing interest

The authors declare that they have no known competing financial interests or personal relationships that could have appeared to influence the work reported in this paper.

## Data Availability

Data will be made available on request.
